# The Sono-Photocatalytic Performance of PAN/g-C_3_N_4_/CdS Nanofibers Heterojunction

**DOI:** 10.3390/ma14205959

**Published:** 2021-10-11

**Authors:** Jing Zhang, Weijie Zhao, Jiaqi Pan, Ruimin Tang

**Affiliations:** 1 Institute of Applied Biotechnology, Taizhou Vocational & Technical College, Taizhou 318000, China; zjzztzmh2018@tzvtc.edu.cn; 2 Key Laboratory of Optical Field Manipulation of Zhejiang Province, and Key Laboratory of ATMMT Ministry of Education, Department of Physics, Zhejiang Sci-Tech University, Hangzhou 310018, China; panjq@zstu.edu.cn; 3 Natural Resources Survey Institute of Heilongjiang Province, Harbin 150036, China

**Keywords:** PAN/g-C_3_N_4_/CdS heterojunction, low power ultrasound, sono-photocatalytic activity, nanofibers

## Abstract

The Polyacrylonitrile (PAN)/g-C_3_N_4_/CdS nanofiber sono-photocatalysts were successfully synthesized by an ordinary electrospining-chemical deposition method. The PAN/g-C_3_N_4_/CdS heterojunction nanofibers constructed with the CdS nanoparticles deposited on the PAN/g-C_3_N_4_ nanofibers. The g-C_3_N_4_/CdS heterojunction increase of light absorption and the construction of heterojunction can depress recombination of charge carrier and PAN nanofibers improve the recyclability successfully. Finally, a highly effective photocatalytic activity was performed by degradation of Rhodamine B (RhB) in visible light irradiation. Furthermore, an ultrasonic method is introduced into the sono-photocatalytic system to enhance the degradation efficiency of RhB ascribed to the synergistic effect of ultrasound.

## 1. Introduction

Semiconductor-based photocatalysts have attracted more and more attention, since Fujishima’s team found water splitting with TiO_2_ in 1972 [1]. Especially in recent years, many promising semiconductor photocatalysts, such as TiO_2_ [2,3,4,5], ZnO [6,7,8] and SnO_2_ [9,10,11,12], have been widely reported. Among those, graphite-like phase carbon nitride (g-C_3_N_4_), with inexpensive, physicochemical stability and suitable potentials, has been extensively used to degrade refractory organic contaminants and hydrogen production as new metal-free semiconductor photocatalysts [1,3,4,5,6,7,8,9,10,11,12,13,14,15]. However, the low sunlight response caused by its intrinsic band gap (2.7 eV), and the strong recombination rate and low mobility of charge carrier have restricted the photocatalytic activity of single g-C_3_N_4_ [16,17,18] seriously. Therefore, many efforts, such as the metal or nonmetal elements doping, surface heterostructures modification, etc., have been tried to improve its catalytic activity. In particular, constructing a heterojunction with another semiconductor obtains a suitable potential, such as g-C_3_N_4_/BiOI [19], LaFeO_3_/g-C_3_N_4_/BiFeO_3_ [20], and g-C_3_N_4_/Ag_3_PO_4_ [21], can depress the recombination of charge carrier, and extend the solar response.

Herein, cadmium sulfide (CdS), with a suitable band gap of 2.42 eV, has been widely reported as a sensitizer to achieve visible light response [22,23,24,25]. Nevertheless, due to the photocorrosion caused by photogenerated holes self-oxidation, CdS would be very unstable during the photocatalytic process [26,27,28]. Actually, the fabrication of heterostructures would drive the holes from CdS transferring to another semiconductor, which is regarded as an effective approach. By calculation, the potentials of g-C_3_N_4_ and CdS obtain a well-matching, which is very suitable to form a high-quality heterojunction to facilitate separation and transfer of photogenerated charge carrier [29,30,31,32].

Nevertheless, poor dispersion and easy aggregation would be another important issue for g-C_3_N_4_/CdS heterojunction, which would result in a significant decrease in photocatalytic efficiency [33]. Meanwhile, the g-C_3_N_4_/CdS heterojunction photocatalysts faced with the problems about separation and reuse, result in the large-scale unrealistic application [34]. In order to solve these problems, a suitable support would be a promising strategy. Polyacrylonitrile (PAN) nanofibers prepared by electrospinning with an over-long one dimensional nanostructure and excellent flexibility are regarded as an ideal support for fixing the heterostructure [35,36]. Therefore, the PAN/g-C_3_N_4_/CdS photocatalysts formed by immobilization of g-C_3_N_4_/CdS on the PAN is more favorable for recycling in large-scale photocatalytic reactions.

Moreover, the catalytic performance is expected to improve further. Recently, a series of literatures have reported that the introduction of ultrasound would be an efficient method. Compared with a single ultrasound or a single photocatalysis, the sono-photocatalysis with synergistic effects will obtain a great improvement during the degradation process [37,38]. Thus, the synergistic effects of ultrasound and photocatalysis is reported as an efficient method for enhancing degradation efficiency in sonophotocatalysis [39]. Additionally, this sonophotocatalysis also can produce a tremendous number of active free radicals in a very short period of time, which are adequate to oxidate the intractable organic pollutants [40].

In the paper, the PAN/g-C_3_N_4_/CdS nanofibers heterojunction were prepared by electrospinning and chemical deposition. The optimum synthesis condition was investigated to prepare PAN/g-C_3_N_4_/CdS heterojunction photocatalysts with excellent photocatalytic performance. The sono-photocatalytic performance was investigated by the degradation efficiency of RhB. Furthermore, the sono-photocatalytic mechanism of PAN/g-C_3_N_4_/CdS nanofibers heterojunction was discussed.

## 2. Methods/Experimental

### 2.1. Chemicals

Melamine (C_3_H_6_N_6_, AR), thiourea (CH_4_N_2_S, AR), N, N-Dimethylformamide (DMF, AR), and cadmium acetate dihydrate (Cd(CH_3_COO)_2_·2H_2_O, AR) were supplied from Aladdin Chemical Co., Ltd. (Shanghai, China). Polyacrylonitrile (PAN, M_W_ = 150,000) was supplied from Sinopharm Chemical Reagent Co., Ltd. (Beijing, China). Chemical reagents were not purified ulteriorly.

### 2.2. Synthesis of PAN/C_3_N_4_/CdS Heterojunction

The bulk g-C_3_N_4_ was synthesized through a process reported in previous literature [41]. As always, 5 g of melamine was put into a covered alumina crucible, warmed to 550 °C at a rate of 5 °C min^−^^1^ in a high temperature furnace, and retained for 4 h. Subsequently cooled to the room temperature, the acquired yellow product was gathered and milled into powder for later use. Then, 1 g of bulk g-C_3_N_4_ was added into 10 mL of sulphuric acid and agitated for 8 h under ordinary temperature. The gained mixed solution was slowly devolved to a certain amount of deionized water and sonicated for exfoliation. During this procedure, it may be observed that the color of gained suspension changed from yellow to light yellow. The suspension was then centrifuged at 6000 rpm for five minutes to eliminate all un-exfoliated g-C_3_N_4_, washed with deionized water for three times to dispose of the remained sulphuric acid, heated to 80 °C, and held for 12 h in the air.

**Scheme 1:**
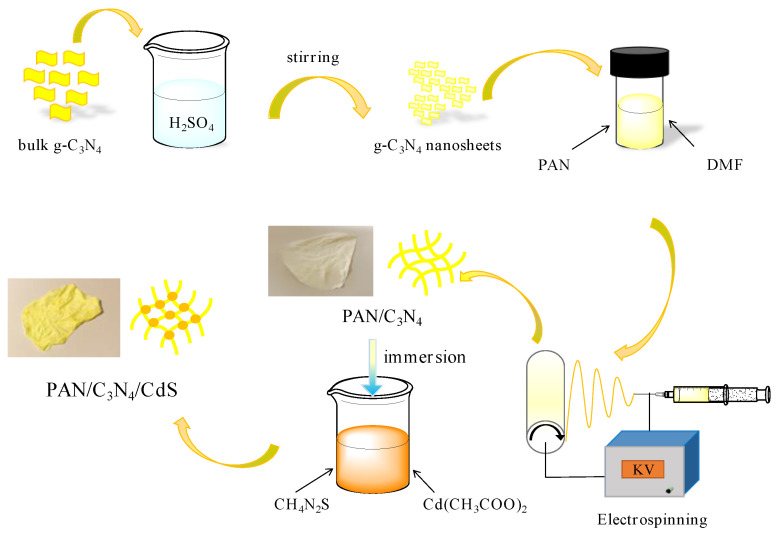
A diagram of the PAN/g-C_3_N_4_/CdS heterojunction preparation.

The PAN/C_3_N_4_/CdS heterojunction was synthesized by electrospinning-chemical deposition method. In brief, 0.1 g of as-synthesized g-C_3_N_4_ was mixed into 8 mL of N, N-Dimethylformamide (DMF) with sonication for 2 h, and then 1 g of polyacrylonitrile (PAN) was placed into the above solution to form a uniform solution after agitating for 2 h. The uniform solution was loaded into an injector for electrospun nanofibers. The process condition of electrospinning was that the working high voltage was set to 15 kv, the injection rate was set to 0.8 mL h^−^^1^, and the receiving distance was set to 15 cm. The obtained PAN/C_3_N_4_ nanofibrous membranes were further warmed to 80 °C and held for 12 h. A certain amount of PAN/C_3_N_4_ nanofibers were dispersed into a mixed solution with the same molar concentration of cadmium acetate and thiourea, and a pH value of the above solution was then controlled to 10 through introducing ammonia water. It was found that the light yellow fibers were transformed to yellow, and the PAN/C_3_N_4_/CdS nanofibers was successfully prepared, which is shown in Scheme 1. The molar concentrations of the mixed solution (cadmium acetate and thiourea) were 0, 0.05, 0.10, and 0.15, which are labelled as PC, PC-Cd-0.05, PC-Cd-0.10, and PC-Cd-0.15.

### 2.3. Characterization

The phase analysis of the PAN/g-C_3_N_4_/CdS nanofibers was investigated through X-ray diffraction (XRD, Bruker D8 diffractometer, Bruker AXS, Karlsruhe, Germany). The micromorphology of the PAN/g-C_3_N_4_/CdS nanofibers was studied via scanning electron microscopy (SEM, Hitachi S-4800, Hitachi High-Technologies Corporation, Tokyo, Japan) and transmission electron microscopy (TEM, JEM-2100, JEOL Ltd., Tokyo, Japan). The UV–vis diffuse reflectance spectra (DRS) was taken on the UV–vis spectrometer (Pgeneral TU-1950, Beijing Purkinje General Instrument Co., Ltd., Beijing, China). The photoluminescence emission spectra (PL) of the PAN/g-C_3_N_4_/CdS nanofibers were recorded with a fluorescence spectrophotometer (Varian Cary Eclipse, λex = 340 nm, Mulgrave, Victoria, Australia) to examine the recombination of photon-generated charge carriers.

### 2.4. Sonophotocatalytic Activity Test

**Figure 1:**
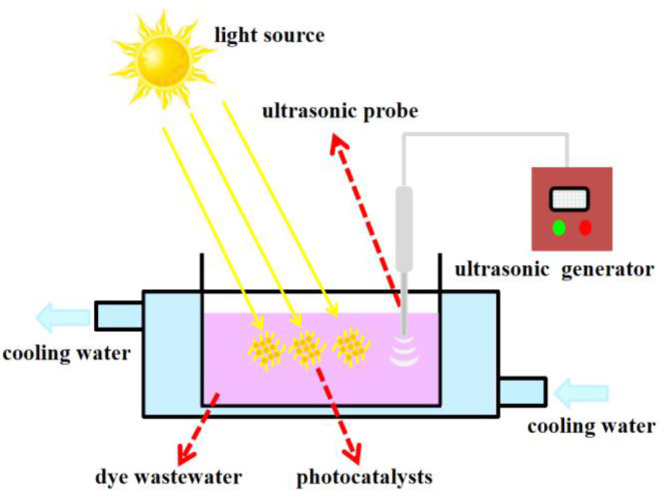
Schematic diagram of the sono-photocatalytic reactor.

As can be seen from the Figure 1, the sono-photocatalytic system is made up of an ultrasonic generator UP250 (Ningbo Scientz Biotechnology Co., Ltd., Ningbo, China), a tungsten-halogen lamp (250 W) using a 420 nm light filter, and a 100 mL double jacket quartz reactor. The sono-photocatalytic property of PAN/C_3_N_4_/CdS heterojunction was investigated through degradation of Rhodamine B in the sono-photocatalytic system. In this experiment, 20 mg PAN/C_3_N_4_/CdS heterojunction samples were added into 100 mL of 10 mg/L Rhodamine B solution and agitated for 30 min in the darkness to reach adsorption-desorption equilibrium between organic substrates and photocatalysts. Then, ultrasound and visible light irradiation were simultaneously performed, and a certain volume of suspension (around 3 mL) was taken and centrifugated at 6000 rpm for 10 min at fixed intervals till the color became colorless. In this process, an external cooling coil was used for water cycling to keep the suspension temperature around 25 °C. Lastly, the concentration of Rhodamine B was confirmed through detecting the maximum absorbance (λmax = 554 nm) employing UV-Vis spectrophotometer (Pgeneral TU-1950, Beijing Purkinje General Instrument Co., Ltd., Beijing, China). The carriers trapping experiments were implemented at uniform conditions through introducing the ammonium oxalate (AO, as trapping agents for h^+^), isopropyl alcohol (IPA, as trapping agents for ·OH), and benzoquinone (BZQ, as trapping agents for ·O_2_^−^), too.

## 3. Results and Discussion

**Figure 2:**
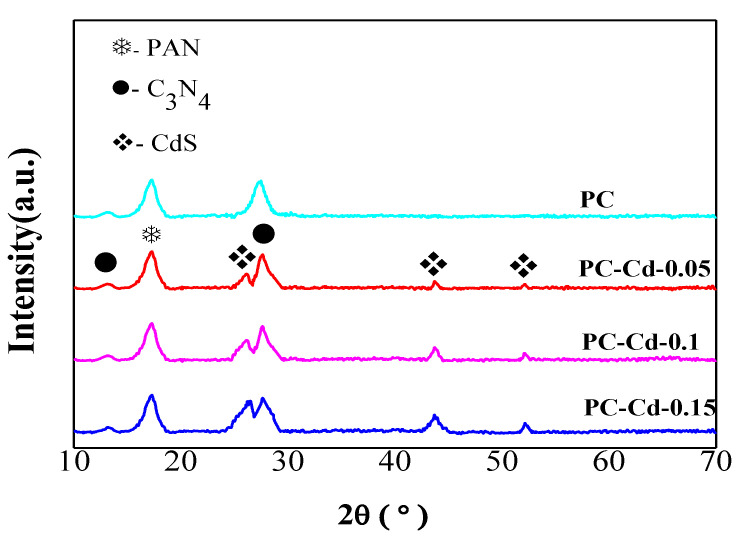
XRD spectra of PAN/g-C_3_N_4_ with different concentration of CdS.

The phase state and structure of as-synthesized PAN/C_3_N_4_/CdS nanofibers heterojunction with various concentration of CdS were investigated through XRD measurements and shown in Figure 2. All samples present three obvious characteristic peaks at 2θ = 13.1°, 27.5°, and 17.1° that are ideally indexed to g-C_3_N_4_ phase (001) and (002) planes (JCPDS-87-1526) [42,43] and PAN phase (111) planes [44], respectively. With the CdS nanoparticles deposition, the samples present three new and obvious characteristic peaks at 2θ = 26.5°, 43.7°, and 52.1° that are well attributed to CdS phase (111), (220) and (311) planes (JCPDS-80-0019) [45]. It was confirmed that the CdS were successfully introduced to PAN/C_3_N_4_ and had formed a composite photocatalyst. With increasing CdS concentration, the corresponding diffraction peaks at 26.5°, 43.7°, and 52.1° exhibit an enhancement. Those above XRD patterns reveal that the samples are made up of PAN, g-C_3_N_4_, and CdS.

**Figure 3:**
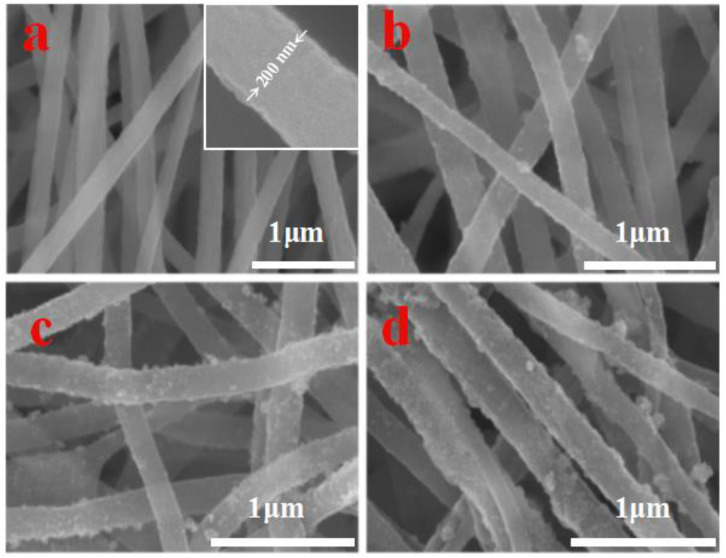
The SEM of (**a**) PC, inset: high resolution SEM, (**b**) PC-Cd-0.05, (**c**) PC-Cd-0.1, and (**d**) PC-Cd-0.15.

The surface morphology and microstructure of as-prepared PAN/g-C_3_N_4_/CdS nanofibers heterojunction were examined with SEM and TEM directly. It is shown in Figure 3a that the obtained PC nanofibers with average diameter approximately 200 nm is uniform, continuous, and rough. The insert is the HRSEM image of the as-prepared nanofibers. As seen, the surface of as-prepared PC sample is rough, which can offer a relatively high surface area to facilitate more CdS nanoparticles growth and increase reaction interfaces for improving the photocatalyst. Figure 3b–d show the different amounts of CdS nanoparticles have been grown on the surfaces of PAN/C_3_N_4_ nanofibers successfully, and it is obvious that the amount of CdS nanoparticles increases with the concentration of the mixed solution.

**Figure 4:**
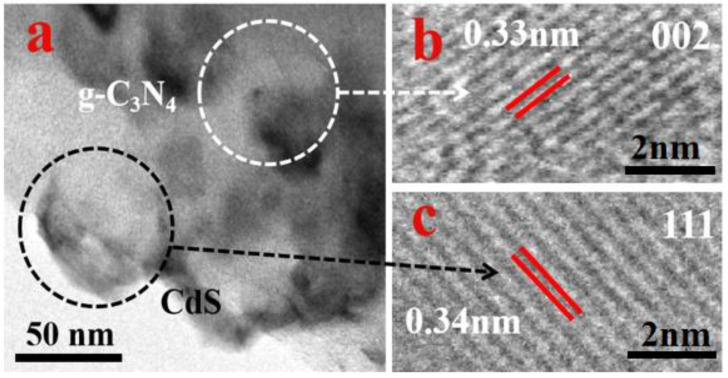
The TEM of PAN/g-C_3_N_4_/CdS heterojunction, (**a**) the TEM of the interface, (**b**) the HRTEM of g-C_3_N_4_, and (**c**) the HRTEM of CdS.

The microstructure information is revealed by the TEM in Figure 4. Figure 4a indicates the CdS nanoparticles are associated with the interface of PAN/g-C_3_N_4_ nanofibers and correspond to the SEM image. Moreover, the high-resolution TEM image of sample is displayed in Figure 4b,c. As the figures show, the lattice spacings of 0.34 nm and 0.33 nm are attributed to (111) facets of CdS [46] and (002) facets of g-C_3_N_4_ [41], severally. This result implies that the CdS has been introduced into PAN/g-C_3_N_4_ nanofiber.

**Figure 5:**
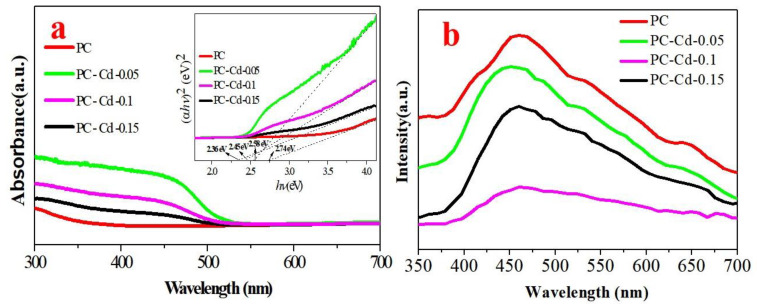
(**a**) The UV–visible spectra of PAN/g-C_3_N_4_/CdS heterojunctions with diverse CdS2 (inset: the corresponding (*αhν*)^2^~*hν* plot), (**b**) The PL spectra of PAN/g-C_3_N_4/_CdS heterojunctions with diverse CdS.

The UV-Vis diffuse reflectance spectra (DRS) of PAN/g-C_3_N_4_/CdS nanofibers heterojunction with diverse proportion of CdS are exhibited in Figure 5a. As illustrated, the slope at approximately 450 nm corresponds to the PAN/g-C_3_N_4_ [34], which is ascribed to the inherent band gap of g-C_3_N_4_ (~2.7–2.9 eV) [47]. Subsequently, the absorption of PAN/g-C_3_N_4_/CdS heterojunction demonstrates an evidently red-shift and improvement in visible light by increasing a certain amount of CdS, which should be ascribed to the inherent band gap of CdS (~2.1–2.4 eV) [47]. As revealed, the red-shift and increased optical absorption are considered as significant factors for the increasement of visible-light photocatalysis. Additionally, the band gaps of the PAN/g-C_3_N_4_/CdS were calculated using the equation: (*αhν*)1/*n* = *A*(*hν* − *Eg*), where *ν* is the vibration frequency, *h* is Planck’s constant, *A* is a proportional constant, *Eg* is the bandgap energy, and *α* is the absorption coefficient. The value of the exponent *n* denotes the nature of the sample transition and is defined as 0.5 for a direct transition semiconductor. The corresponding (*αhν*)^2^~*hν* plot for the PAN/g-C_3_N_4_/CdS is shown in the inset of Figure 5a. The fitting results indicate that the band gap of PC, PC-Cd-0.05, PC-Cd-0.1, and PC-Cd-0.15 is approximately 2.74 eV, 2.58 eV, 2.45 eV, and 2.36 eV, respectively.

The PAN/g-C_3_N_4_/CdS heterojunction of photoinduced interfacial charge transfer processes is researched by photoluminescence spectroscopy (PL). All samples show unique PL signals by excitation at 340 nm. According to the Figure 5b, the impact of CdS is obviously demonstrated by the remarkably decreased PL spectra than that PAN/g-C_3_N_4_ nanofiber. Hence the PAN/g-C_3_N_4_/CdS heterojunction is intended to show lower recombination between photogenerated charge carrier, because the lower PL spectra intensity implies a stronger separation of photogenerated charge carrier. In this PAN/g-C_3_N_4_/CdS heterojunction, different potentials exist between CdS and g-C_3_N_4_, therefore boosting the movement of photo-generated electrons (e^−^) from CB (g-C_3_N_4_) to CdS can minimize the recombination possibility of photogenerated charge carrier efficiently. For all as-prepared samples, the PC obtains the highest PL spectra, and implies its highest recombination tendency, which can be attributed to the absence of CdS, would result in a weak effect of photogenerated electron-hole pairs separation. The PC-Cd-0.05, PC-Cd-0.1, and PC-Cd-0.15 contained different ratios of CdS, which have formed the PAN/g-C_3_N_4_/CdS heterojunctions, which can improve the migration of photogenerated charge carrier to decrease the PL. Moreover, it is obvious that the PC-Cd-0.1 obtained the lowest PL, which manifests that the separation of photogenerated charge carrier is maximized.

**Figure 6:**
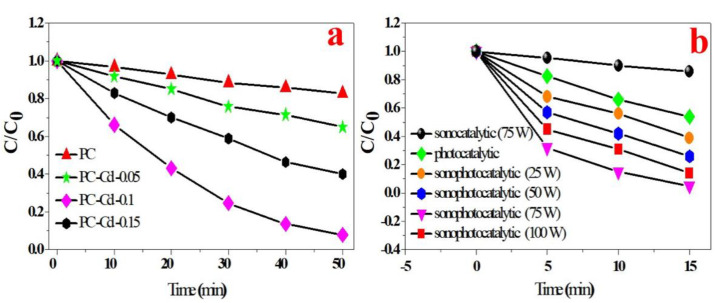
(**a**) Photocatalytic degradation curves of PAN/g-C_3_N_4_/CdS heterojunctions. (**b**) Sono-photocatalytic degradation curves of PAN/g-C_3_N_4_/CdS heterojunctions (PC-Cd-0.1).

The photocatalytic, sonocatalytic, and sono-photocatalytic activity of degradation of RhB are investigated in Figure 6. As shown in Figure 6a, the degradation rate of PC is weak (18.0%, under visible light irradiation for 50 min), while the degradation efficiency improves obviously with the increased CdS and obtains an optimal value at the PC-Cd-0.1 (92%), then decreases. By calculating, the degradation efficiency of PC-Cd-0.1 is about five times that of PC. One of the main reasons is attributed to the constructed heterojunction of PAN/g-C_3_N_4_/CdS, which can facilitate the separation of photogenerated charge carrier efficiently, which is beneficial to the photocatalytic activity. Figure 6b exhibits the degradation efficiency of sono-photocatalysis (PC-Cd-0.1). As revealed, the introducing of ultrasound can improve the degradation efficiency of PC-Cd-0.1 effectively, which shows marked improvement over that of single photocatalytic system and single sonocatalytic system. When the ultrasonic power is 75 W, the highest degradation efficiency of sono-photocatalytic system was 92% within 15 min, which is almost twice that of a single photocatalytic system and seven times that of a single sonocatalytic system.

**Figure 7:**
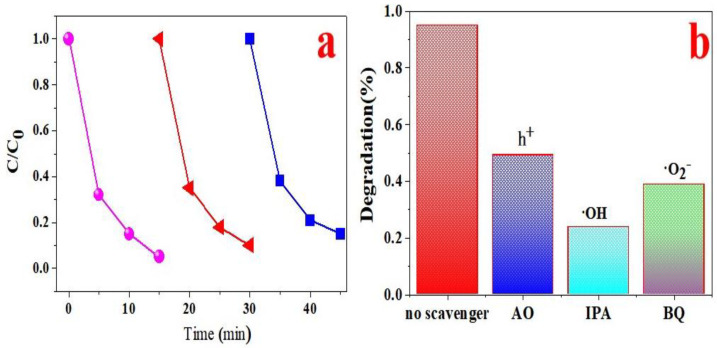
(**a**) PAN/g-C_3_N_4_/CdS cyclic stability to RhB degradation in sono-photocatalytic process. (**b**) The diverse carrier trapping on the RhB degradation for the PC-Cd-0.1 under the sono-photocatalytic system with a 75 W ultrasonic power.

The lifetime and reusability of the pholocatalysts are very important for the practical application of sono-photocatalytic reactions. Figure 7a shows little decrease in the degradation capacity of the PAN/g-C_3_N_4_/CdS heterojunction photocatalysts after three sono-photocatalytic processes, indicating that the PAN/g-C_3_N_4_/CdS heterojunction photocatalysts has very good stability and also predicting promising prospects for future practical applications of sono-photocatalytic processes.

The stability of PAN/g-C_3_N_4_/CdS heterojunctions in sono-photocatalytic process of acoustic to degrade RhB decreases little after three cycles, further indicating that the stability of photocatalysts during acoustic to photocatalysis is excellent, which will be promising for future practical applications of processing RhB.

Free-radical trapping experiments are used for discussing the mechanism of sono-photocatalytic degradation of RhB with regard to PAN/g-C_3_N_4_/CdS heterojunctions (PC-Cd-0.1) as a photocatalyst. Using diverse trapping agents to investigate carrier trapping of PC-Cd-0.1 in sono-photocatalytic system is shown in Figure 7b. As displayed, AO (trapping agents for h^+^) and BZQ (trapping agents for ·O_2_^−^), as trapping agents of free radicals, all exhibit a relatively minor effect, which illustrates that h^+^ and ·O_2_^−^ are not the most important factors for the sono-photocatalytic system with PC-Cd-0.1. Interestingly enough, IPA (trapping agents for ·OH) shows the strongest effect, indicating that ·OH is the real protagonist of sono-photocatalytic system for degradation Rhodamine B.

**Figure 8:**
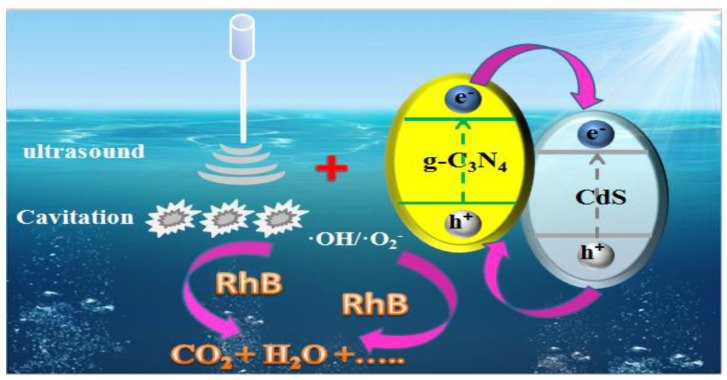
Proposed sono-photocatalytic mechanism for degradation of RhB with PAN/g-C_3_N_4_/CdS heterojunction.

The possible mechanism concerning the sono-photocatalytic system degradation of RhB by PAN/g-C_3_N_4_/CdS heterojunction photocatalysts is proposed in Figure 8. As revealed, bubbled with high temperatures and pressures (generated through ultrasound cavitation in dye wastewater), this system can generate extremely active free radicals, such as ·OH and ·H. Subsequently, H takes a combination with O_2_ to produce ·OOH free radicals. Both ·OH and ·OOH radicals can degrade RhB effectively [38]. On the other hand, after the CdS is added into the PAN/g-C_3_N_4_, the formed heterojunction is beneficial to boost the fast separation of photogenerated charge carrier efficiently. Such process is depicted as follows. Through visible light irradiation, both CdS and g-C_3_N_4_ are excited and produce photogenerated charge carrier. Owing to the well-matched potentials and closely contacted interfaces of the g-C_3_N_4_/CdS heterojunction, the electrons (e^−^) on the CB (g-C_3_N_4_) can quickly migrate to the CB (CdS), and meanwhile, the holes (h^+^) on the VB (CdS) rapidly migrate to the VB (g-C_3_N_4_), which is conducive to promoting the photogenerated charge carrier separation effectively, and is beneficial for enhancing sono-photocatalytic activity [48].

Further, a credible pathway of sono-photocatalytic oxidation of RhB is proposed as follows:

Ultrasound + H_2_O → ·H+ ·OH (1)

·H + O_2_ → ·OOH (2)

PAN/g-C_3_N_4_/CdS + hν → h^+^ + e^−^ (3)

h^+^ + OH^−^ → ·OH (4)

h^+^ + H_2_O → ·OH +·H (5)

O_2_ + e^−^
→ ·O_2_^−^ (6)

·OH + RhB → CO_2_ + H_2_O + Others (7)

·O_2_^−^ + RhB→ CO_2_ + H_2_O + Others (8)

h^+^ + RhB → CO_2_ + H_2_O + Others (9)

## 4. Conclusions

In summary, an ideal design the PAN/g-C_3_N_4_/CdS heterojunction photocatalysts has been successfully synthesized by simple processes and proved to be highly effective for the degradation of RhB in sono-photocatalytic system. The main reason can be attributed to the effective synergy between the ultrasound and photocatalysis. In addition, the PAN/g-C_3_N_4_/CdS heterojunction also plays an important role during the process of sono-photocatalysis. Herein, the PAN/g-C_3_N_4_/CdS heterojunction composite photocatalysts formed by CdS deposited on the PAN/g-C_3_N_4_ can not only increase optical absorption, but also depress the recombination of photogenerated charge carrier. Therefore, the sono-photocatalytic system of PAN/g-C_3_N_4_/CdS heterojunction can degrade RhB up to 92% within 15 min. Meanwhile, the experiment data indicate that the different scavengers would directly affect the degradation efficiency of the sono-photocatalytic system. In the degradation of RhB, the generated hydroxyl radicals would play a more important role than the superoxide radicals and photo-generated holes, and a general order can be proposed as: ·OH > ·O_2_^−^ > h^+^.

## Data Availability

All data are contained within the manuscript.
